# The Clinical Implication of Conversion Surgery in Patients with Stage IV Gastric Cancer Who Received Systemic Chemotherapy

**DOI:** 10.3390/biomedicines11113097

**Published:** 2023-11-20

**Authors:** Min-Kyue Shin, Min-Gew Choi, Seung-Tae Kim, Won-Ki Kang, Tae-Sung Sohn, Ji-Yeong An, Joon-Ho Lee, Jee-Yun Lee

**Affiliations:** 1 Division of Hematology-Oncology, Department of Medicine, Samsung Medical Center, Sungkyunkwan University School of Medicine, Seoul 06351, Republic of Koreawonki.kang@samsung.com (W.-K.K.); 2 Department of Digital Health, Samsung Advanced Institute for Health Sciences & Technology, Sungkyunkwan University, Seoul 06355, Republic of Korea; 3 Department of Surgery, Samsung Medical Center, Sungkyunkwan University School of Medicine, Seoul 06351, Republic of Korea

**Keywords:** stomach neoplasms, gastrectomy, neoadjuvant therapy, molecular targeted therapy

## Abstract

With the advances in chemotherapy and immunotherapy, a small subset of patients may be eligible for conversion surgery after achieving tumor regression with chemotherapy. This is a retrospective cohort study of 118 patients with stage IV gastric cancer who received palliative chemotherapy and conversion surgery with a negative resection margin at Samsung Medical Center. Baseline features included comorbidities, body mass index (BMI), carcinoembryonic antigen (CEA) level, primary tumor size, biopsy histology, distant metastatic sites, and molecular markers—HER2, MSI/MMR, PD-L1, and EBV. Post-chemotherapy features included BMI, CEA level, chemotherapy regimen, objective response to chemotherapy, and number of preoperative chemotherapy cycles. Post-operational features included tumor size, histologic differentiation and Lauren’s classification, pathologic tumor and nodal stages, invasion of lymphatics/vessels/nerves, peritoneal cytology, and the receipt of postoperative chemotherapy. Of 118 patients, 60 patients received total gastrectomy and 58 patients received subtotal gastrectomy. In all, 21 patients achieved a pathologic complete response, and 97 patients achieved downstaging to yp stage I, II, or III. Before conversion surgery, patients received first-line capecitabine/oxaliplatin (62%), HER2 inhibitors combined with chemotherapy (18%), immune checkpoint inhibitors (15%), and inhibitors of MET or VEGFR2 (5%). In the multivariable analysis, BMI at the time of diagnosis, either HER2 positive, high MSI, or deficient MMR, and the use of targeted agents were significant prognostic factors. Conversion surgery could be considered in patients with stage IV gastric cancer regardless of the initial disease burden. BMI and molecular markers are important prognostic factors that can be used to select candidates.

## 1. Introduction

The prognosis of stage IV gastric cancer patients is still poor; median overall survival (OS) remains a little bit longer than one year, notwithstanding the addition of targeted agents to cytotoxic chemotherapy [1,2]. A wealth of studies suggest that the curative gastrectomy could improve clinical outcomes in these patients, including those with positive peritoneal cytology, peritoneal metastasis, extensive lymph node metastasis, or liver metastasis [3,4,5,6,7,8,9,10,11]. Although a phase 3 randomized controlled trial (REGATTA) demonstrated no survival benefit of gastrectomy followed by chemotherapy over chemotherapy alone in gastric cancer patients with a single non-curable factor (either liver, peritoneum, or para-aortic lymph nodes) [12], its study design is different from the majority of the aforementioned studies, which investigated the efficacy of gastrectomy following preoperative chemotherapy [5,6,7,9,10,11]. So-called “conversion surgery” benefits from higher R0 resection and pathological response rates, as well as the clearance of free cancer cells in the peritoneal cavity. Indeed, retrospective analyses, including a meta-analysis and nationwide analysis, demonstrated the survival benefit of conversion surgery compared to primary gastrectomy, plus postoperative chemotherapy or chemotherapy alone [13,14,15]. In addition, a prospective phase 2 trial (AIO-FLOT3) had a median OS of 22.9 months for patients with limited metastatic gastric cancer treated via conversion surgery [16]. More recently, a retrospective Asian cohort study (CONVO-GC-1) reported median OS of 36.7 months for 1206 stage IV gastric cancer patients who received conversion surgery [17]. Furthermore, a phase 3 trial (RENAISSANCE/AIO-FLOT5) of conversion surgery is ongoing for patients with retroperitoneal lymph node metastases with or without a single incurable organ site [18].

Given the importance of the proper selection of candidates for conversion surgery aimed at curative R0 resection, recent studies on conversion surgery focused on factors associated with survival [14,15,17,19,20,21,22,23,24,25]. Multivariable analyses identified several prognostic factors that can be divided by their timing of clinical acquisition [19,20,22,23,24]. Firstly, Eastern Cooperative Oncology Group (ECOG) performance status, ascites, and the number of incurable factors were identified before the initiation of chemotherapy. Secondly, the number of cycles of first-line chemotherapy, response to chemotherapy, and the change in carcinoembryonic antigen (CEA) levels were measured prior to surgery. Lastly, the gastrectomy type (partial vs. total), achievement of R0 resection, and receipt of adjuvant chemotherapy were confirmed after surgery. Based on this temporal classification, which is important regarding the choice of treatment strategy, in this study, we aimed to identify prognostic factors in stage IV gastric cancer patients treated with R0 conversion surgery. We limited our analysis to R0-resected patients due to the significant impact on survival—the median OS for 839 patients of R0 resection was 56.6 months in the CONVO-GC-1 study [17]—and variability between hospitals.

## 2. Materials and Methods

**Figure 1:**
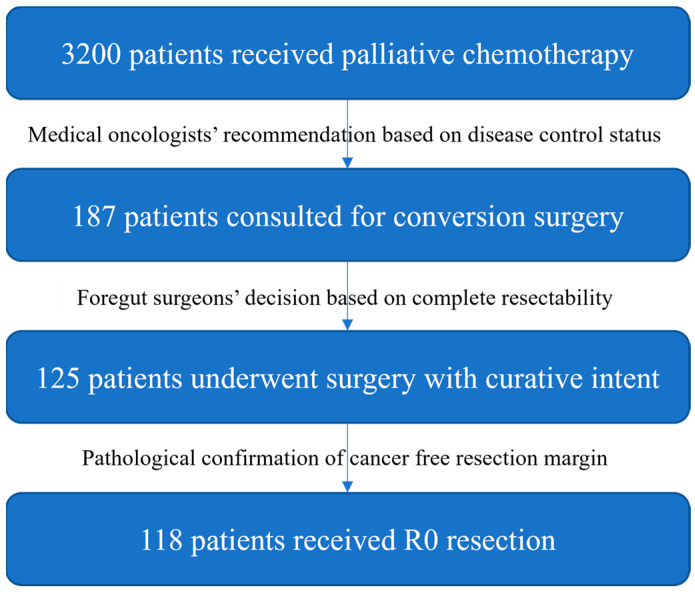
Patient selection flow chart.

Between January 2016 and October 2022, 3200 patients received palliative chemotherapy for metastatic gastric cancer at Samsung Medical Center. If a patient’s disease seemed to be controlled according to the computed tomography (CT) images checked after chemotherapy, and the patient’s general condition (including performance status and comorbidity) was thought to be good enough for the operation, a physician would recommend a consultation for conversion surgery if the patient agreed. Of 187 patients consulted for potential conversion surgery, 125 patients underwent surgery with curative intent and 118 patients achieved R0 resection (Figure 1). The inclusion criteria were as follows: (1) histologically confirmed primary gastric cancer, (2) clinical diagnosis of stage IV disease according to the AJCC 7th or 8th staging system, (3) patients who underwent systemic chemotherapy prior to surgery, (4) patients who underwent subsequent gastrectomy with curative intent plus metastasectomy if needed, and (5) the provision of written informed consent. The exclusion criteria were as follows: (1) presence of carcinoma at the resection margin; (2) patients who underwent emergent and/or palliative gastrectomy due to obstruction, bleeding, or perforation; (3) patients who could not discharge after surgery and died due to postoperative complications; (4) incomplete clinical data obtained from the hospital in which previous treatment was carried out; and (5) unavailable treatment data due to a blinded clinical trial. R0 resection was performed in following circumstances: (1) patients whose metastasis showed complete response in imaging, (2) no gross peritoneal seeding was identified by laparoscopic examination, (3) frozen biopsy revealed complete regression in metastasis, and (4) metastasectomy was performed.

Primary tumor size was measured in initial CT and esophagogastroduodenoscopy (EGD), and its histological classification and differentiation were diagnosed using EGD biopsy. Distant metastatic sites were identified from initial abdomen and pelvis CT with or without chest CT. HER2 positivity was defined as either immunohistochemistry (IHC) 3+ or 2+ with fluorescence in situ hybridization HER2:CEP17 ratio ≥2. HER2 IHC was performed with monoclonal rabbit PATHWAY anti-HER2/neu (Ventana). PD-L1 IHC was performed using monoclonal mouse anti-PD-L1, clone 22C3 (Agilent Technologies, Santa Clara, CA, USA) using DAB IHC Detection kit on BenchMark Ultra (Ventana). PD-L1 combined positive score (CPS) was calculated by summing the number of PD-L1-stained cells (tumor cells, lymphocytes, and macrophages) and dividing by the total number of viable tumor cells, then multiplying by 100. EBV status was determined by EBV-encoded small RNA in situ hybridization. Microsatellite instability (MSI) status was identified from TruSight Oncology 500 assay (Illumina, San Diego, CA, USA) or polymerase chain reaction of five quasimonomorphic mononucleotide-repeat markers (BAT25, BAT26, NR21, NR24, and NR27) analyzed on the 3130xL Genetic Analyser (Applied Biosystems, Waltham, MA, USA). DNA mismatch repair (MMR) status was identified from IHC performed with anti-MLH1, clone ES05 (Leica Biosystems, Wetzlar, Germany). Surgical pathologic reports included tumor size, histologic differentiation, Lauren’s classification, and ypTN stage, and the presence of the invasion of lymphatics/vessels/nerves was reported using surgical specimens. Peritoneal cytology was identified from washing fluid obtained during surgery. The latest values of CEA level and body mass index (BMI) were obtained before chemotherapy and conversion surgery, respectively. The last chemotherapeutic regimen, the best response to the corresponding regimen, and comorbidities diagnosed before surgery were collected from electronic medical records. Treatment response was evaluated according to RECIST 1.1.

OS and recurrence-free survival (RFS) were defined as the period from the date of diagnosis to the date of death and recurrence, respectively. Kaplan–Meier curves with log-rank test were drawn using the “survival” and “ggplot2” R libraries to compare OS and RFS between subgroups. Patients who did not experience the event of interest were censored at the date of last follow-up. The “survivalAnalysis” R library was used to fit Cox proportional hazards regression models and draw forest plots. The “rms” R library was used to fit a logistic regression model and draw a nomogram. All statistical analyses were conducted using R version 4.2.2 (R Foundation for Statistical Computing, Vienna, Austria).

## 3. Results

### 3.1. Cohort Description

The clinicopathological characteristics of 118 stage IV GC patients, who received gastrectomy and were confirmed for a resection margin free from carcinoma, are described in Table 1 and Supplementary Table S1. Median age was 56.5 years (range: 32–82) and proportion of male patients was 75%. Median tumor size was 6–7 cm (range: 1.5–15.0) measured at initial EGD or CT, and 3.6 cm (range: 0–18) in the specimen acquired from conversion surgery. The median levels of carcinoembryonic antigen (CEA) were 1.6 ng/mL (range, 0.2–1000) before chemotherapy and 2.0 ng/mL (range: 0.4–64.9) before surgery. HER2 status was positive in 22% of patients, and EBV status was positive in 6%. Of 118 patients, PD-L1 CPS was tested in 44 patients and positive in 35 patients. MSI and/or MMR status was tested in 78 patients and six patients demonstrated high MSI (MSI-H) or deficient MMR (dMMR). Median OS and RFS were 2396 days (range: 305–2907) and 1274 days (range: 141–2907), respectively. OS rates for 1-, 3-, and 5-year survival were 98.3%, 71.1%, and 54.9%. RFS rates for 1-, 3-, and 5-year survival were 83%, 54.8%, and 43.9%.

Before conversion surgery, patients received cytotoxic agents alone (mostly XELOX or FOLFOX) in 62%, capecitabine/cisplatin or XELOX + HER2 inhibitors in 18%, ICIs with or without XELOX in 15%, and inhibitors of MET or VEGRF2 in 5% (detailed information on regimens available in Table S2). While 105 (89.0%) patients were candidates for conversion surgery after first-line cytotoxic chemotherapy with or without ICIs and/or HER2 inhibitors, the other 13 (11.0%) patients achieved adequate tumor regression in further lines of therapy, and 9 of them were assigned by specific biomarkers—2 with HER2+, 1 with MSI-H, 1 with dMMR, 1 with EBV+/PD-L1+, and 4 with MET amplifications. The best objective response to chemotherapy was radiologic complete response in 4%, partial response in 71%, and stable disease in 25% of patients. The depth of invasion identified in surgical specimens was ypT0 in 20%, ypT1 in 14%, ypT2 in 11%, ypT3 in 30%, and ypT4 in 25% of patients. The extent of metastasis to lymph nodes was ypN0 in 49%, ypN1 in 16%, ypN2 in 14%, and ypN3 in 20% of patients. At the study cutoff date (29 December 2022), 54 patients relapsed, and 42 patients died. Frequent locations of recurrences were the peritoneum, distant lymph nodes, retroperitoneal organs, and gastrectomy sites (Table S3).

### 3.2. Univariable Analysis

**Figure 2:**
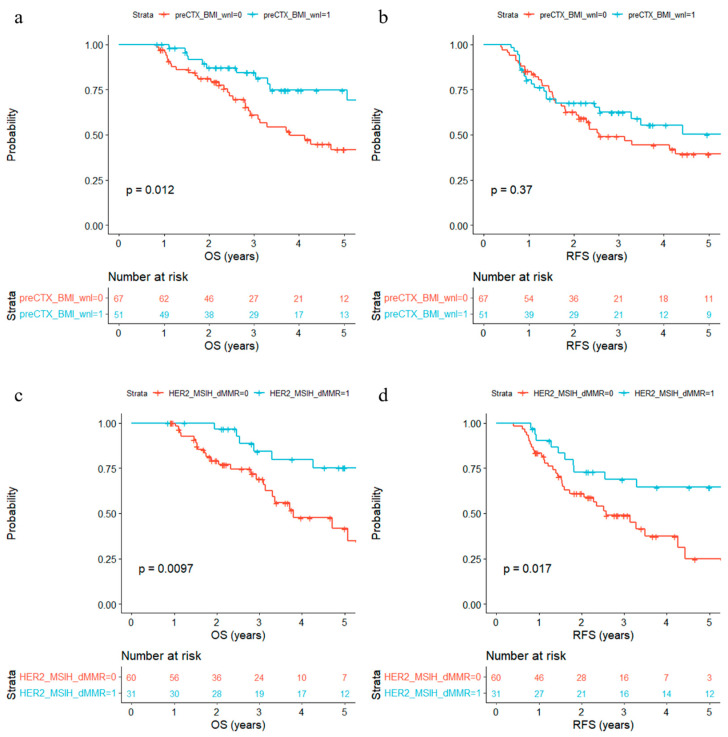
Kaplan–Meier estimates of overall survival (OS) and recurrence-free survival (RFS) according to baseline features. (**a**,**b**) BMI before chemotherapy is (1) or is not (0) within normal limits (greater than 18.5, less than 23.0). (**c**,**d**) Presence (1) or absence (0) of either HER2+, MSI-H, or dMMR.

Next, we tested associations of baseline factors with OS and RFS. BMI within normal limits (18.5–23.0) before chemotherapy was associated with significantly (*p* = 0.012) longer OS and numerically longer RFS (Figure 2a,b), while normal BMI before surgery (*p* = 0.088) showed a similar trend but did not reach significance for OS (Figure S1a). Intriguingly, initial BMI was a significant prognostic factor in HER2- tumors (*p* = 0.0011) but was not in HER2+ tumors (*p* = 0.89) (Figure S2). The presence of comorbidities was not associated with survival, nor was ECOG performance status (Figure S1b,c). Interestingly, the presence of either HER2+, MSI-H, or dMMR was significantly associated with longer OS (*p* = 0.0097) and RFS (*p* = 0.017) (Figure 2c,d). EBV+ or PD-L1+ patients showed a trend of favorable prognosis, but the association was not significant (Figure S1d,e). Tumor size measured in CT (*p* = 0.0441) was significantly associated with prognosis, while tumor size measured in EGD (*p* = 0.0666) demonstrated a greater hazard ratio but did not achieve significance (Table 2). In addition, the pathology of EGD biopsy (*p* = 0.11) was not significantly associated with prognosis, though poorly differentiated adenocarcinoma or signet ring cell carcinoma showed a trend of poor survival (Figure S1f). Furthermore, a metastatic pattern was not associated with prognosis, but patients with multiple metastases had a higher 5-year OS rate (79%) than others (49%) (Figure S3).

**Figure 3:**
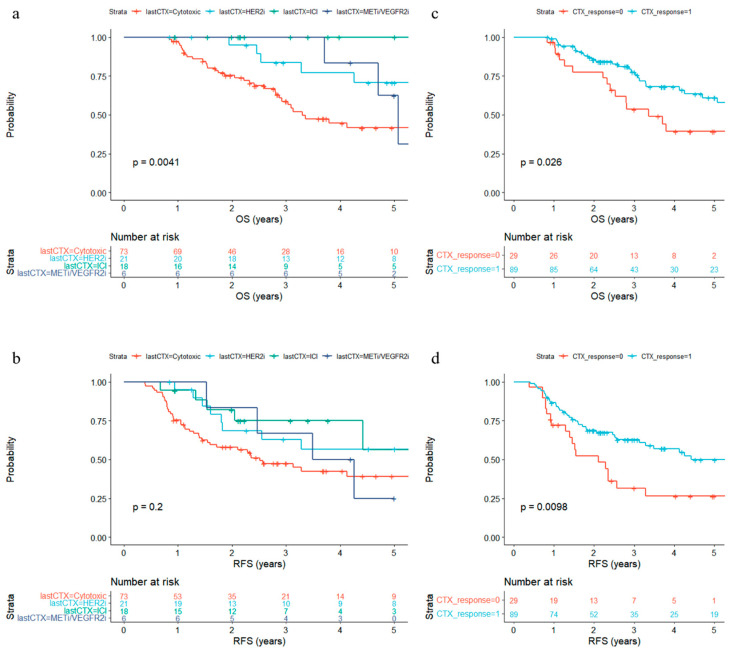
Kaplan–Meier estimates of overall survival (OS) and recurrence-free survival (RFS) according to post chemotherapy features. (**a**,**b**) The last chemotherapy regimen before surgery is classified into cytotoxic agents only (cytotoxic), HER2 inhibitors with or without cytotoxic agents (HER2i), including an immune checkpoint inhibitor (ICI), or including an inhibitor of MET. (**c**,**d**) Achievement of objective response to the last chemotherapy regimen.

Then, we checked associations between post-chemotherapy factors with OS and RFS. The chemotherapy regimen before surgery was significantly (*p* = 0.0041) associated with OS and a similar trend was seen for RFS—cytotoxic agents alone with poor prognosis (Figure 3a,b). Patients who exhibited objective responses (complete or partial response) to chemotherapy before surgery lived significantly longer (*p* = 0.026 for OS and *p* = 0.0098 for RFS) (Figure 3c,d). In total, 24 out of 26 patients with HER2+ tumors and 18 out of 18 patients treated with ICIs showed objective responses to chemotherapy. Moreover, CEA level before surgery (*p* = 0.0191) was significantly associated with survival, while CEA level before chemotherapy (*p* = 0.3389) was not significant (Table 2). In patients with HER2- tumors, preoperative CEA level (*p* = 0.0351) was still a significant prognostic factor, but response to chemotherapy (*p* = 0.12) was not a significant prognostic factor. Moreover, the number of pre-operative chemotherapy cycles (3–4 vs. 5 or greater) was not associated with prognosis, nor was the receipt of postoperative chemotherapy (Figure S4).

**Figure 4:**
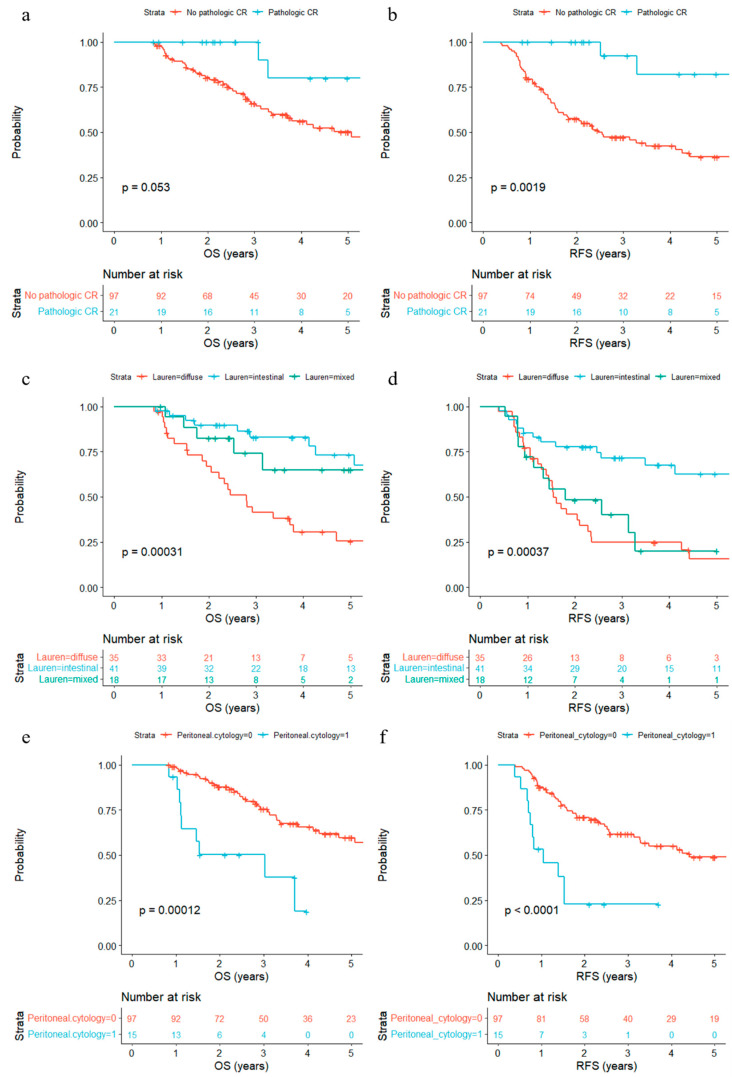
Kaplan–Meier estimates of overall survival (OS) and recurrence-free survival (RFS) according to post operative features. (**a**,**b**) Confirmation of pathologic complete response (CR). (**c**,**d**) Lauren’s classification comparing diffuse vs. mixed vs. intestinal types. (**e**,**f**) Positivity of cytology from peritoneal washing fluid obtained during surgery.

We also examined associations between surgical findings and OS/RFS. Patients who received partial gastrectomy survived longer than patients who received total gastrectomy (*p* = 0.013) (Figure S5a). Pathologic tumor (ypT) and nodal (ypN) stages were significantly associated with prognosis (*p* < 0.0001) (Table 2). Additionally, histological differentiation (*p* = 0.0085), the presence of lymphatic invasion (*p* < 0.0001), venous invasion (*p* = 0.028), and perineural invasion (*p* < 0.0001) were significantly associated with poor prognosis (Figure S5b–e). Patients with pathologic complete response (ypT0N0) showed a favorable trend of OS and significantly longer RFS (*p* = 0.0019) (Figure 4a,b). Lauren’s classification was significantly associated with both OS (*p* = 0.0003) and RFS (*p* = 0.0004)—diffuse and mixed types with poor prognosis (Figure 4c,d). Positive peritoneal cytology from peritoneal washing during surgery (*p* = 0.0001) was also significantly associated with poor prognosis (Figure 4e,f).

### 3.3. Multivariable Analysis

**Figure 5:**
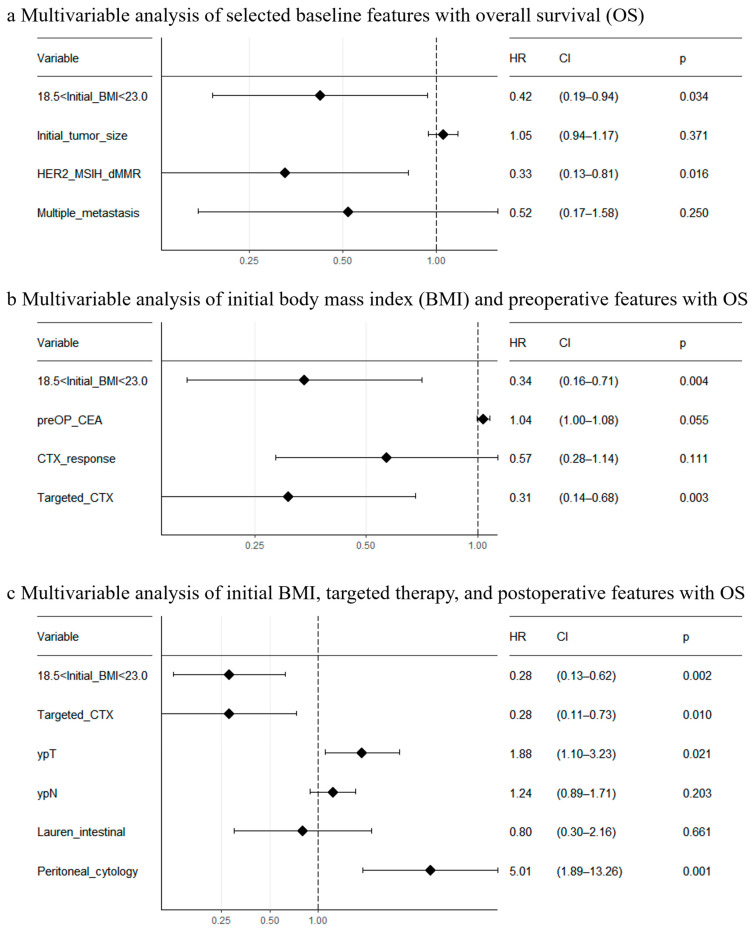
Multivariable Cox proportional hazards regression analysis for overall survival. HR, hazard ratio; CI, confidence interval. (**a**) Variables include whether BMI before chemotherapy is normal (greater than 18.5; less than 23.0), initial tumor size measured by CT (as a continuous variable), either HER2+, MSI-H, or dMMR, and presence of multiple metastases. (**b**) Variables include whether BMI before chemotherapy is normal, CEA level measured before surgery (as a continuous variable), achievement of objective response to the last chemotherapy regimen before surgery, and whether the chemotherapy regimen includes a targeted agent. (**c**) Variables include whether BMI before chemotherapy is normal, whether the chemotherapy regimen includes a targeted agent, pathologic tumor (ypT) and nodal (ypN) stages (as continuous variables), Lauren’s classification (intestinal vs. diffuse/mixed), and positivity of peritoneal cytology.

**Figure 6:**
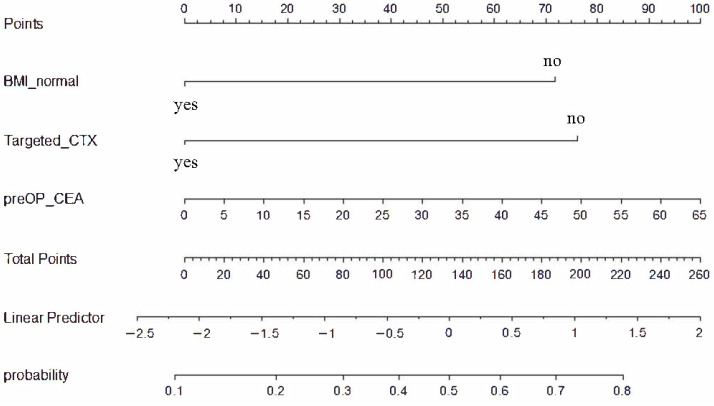
Nomogram of a logistic regression model for the prediction of death. Variables include whether BMI before chemotherapy is normal, whether the preoperative chemotherapy regimen includes a targeted agent, and the CEA level (ng/mL) measured before surgery.

Finally, we performed multivariable analyses for OS using significant variables identified at each clinical stage. Among factors identified at diagnosis, normal BMI (*p* = 0.034) and either HER2+, MSI-H, or dMMR (*p* = 0.016) were significantly associated with favorable prognosis (Figure 5a). Among factors identified prior to surgery, excluding either HER2+, MSI-H, or dMMR due to its significant correlation with the receipt of targeted therapy (Pearson’s Chi-squared test, *p* < 0.0001), normal BMI (*p* = 0.004) and receipt of targeted therapy (*p* = 0.003) were significantly associated with favorable prognosis (Figure 5b). For this analysis, we included both CEA level before surgery and objective response to chemotherapy because they were not significantly correlated (Figure S6). Among the factors identified pre- and post-operation—excluding histologic differentiation due to its significant correlation with Lauren’s classification (Pearson’s Chi-squared test, *p* < 0.0001), method of surgery (Pearson’s Chi-squared test, *p* = 0.0132), tumor size from surgical specimen (analysis of variance, *p* < 0.0001) due to its significant correlation with ypT stage, and lymphatic/venous/perineural invasion due to its significant correlation with ypN stage (Table S4)—normal BMI (*p* = 0.002), receipt of targeted therapy (*p* = 0.010), ypT stage (*p* = 0.021), and positive peritoneal cytology (*p* = 0.001) were significantly associated with survival (Figure 5c). In addition, we constructed a nomogram for the prediction of death using preoperative factors, in order to help the selection of candidates for conversion surgery (Figure 6).

## 4. Discussion

**Figure 7:**
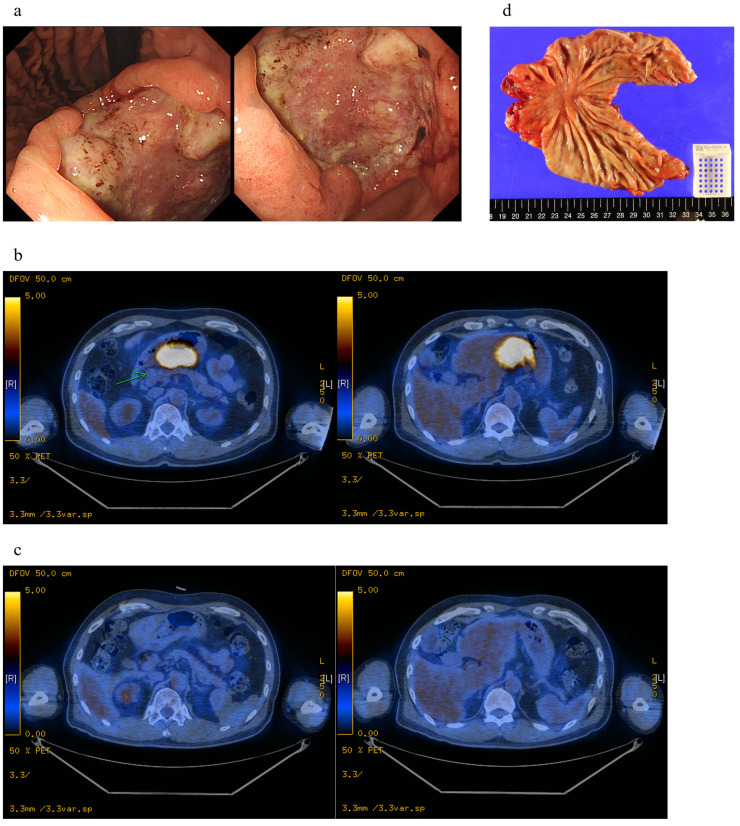
Case presentation of a pathologic complete remission. (**a**) Images from gastroscopy at diagnosis. (**b**) PET-CT image at diagnosis. (**c**) PET-CT image after 11 cycles of chemotherapy (capecitabine + oxaliplatin + pembrolizumab). (**d**) Surgical specimen from gastrectomy after chemotherapy.

In this study, among 118 patients who received conversion surgery, 21 patients achieved pathologic complete remission (pCR) and 97 patients achieved dramatic down staging from stage IV to stages I, II, or III. The likelihood of achieving pCR was significantly higher in patients who received targeted agents or ICIs for specific biomarkers (Pearson’s Chi-squared test, *p* = 0.0030). Especially, pCR was frequently demonstrated in 7 out of 23 HER2 + patients who received HER2-targeted agents and two out of three MSI-H patients who received immunotherapy. Surprisingly, all of the patients treated with preoperative ICIs in our cohort survived during the follow up period (median: 970 days) (Figure 3a). A case presentation is shown in Figure 7. The patient was diagnosed with stage IV gastric cancer with direct invasion to liver (Figure 7a,b) and received capecitabine + oxaliplatin + pembrolizumab. After 11 cycles, PET-CT demonstrated a dramatic shrinkage in the tumor (Figure 7c), and the patient received subtotal gastrectomy. The tumor specimen showed pathologic complete remission (Figure 7d). This is in agreement with results from the KEYNOTE trials, which demonstrate survival benefit of pembrolizumab in MSI-H patients regardless of the number of previous chemotherapy regimens [26]. An interim analysis of the phase 3 MATTERHORN trial (NCT04592913) also showed a significant improvement of pCR in patients with stage II-IVA gastric cancer treated with neoadjuvant durvalumab plus chemotherapy [27]. Moreover, the addition of pembrolizumab to trastuzumab plus cytotoxic chemotherapy significantly increased the objective response rate (ORR) from 52% to 74% in HER2+ patients [28]. Furthermore, trastuzumab deruxtecan—an antibody–drug conjugate consisting of an anti-HER2 antibody, a cleavable linker, and a cytotoxic topoisomerase I inhibitor—significantly improved ORR from 14% to 51% in previously treated HER2+ patients [29]. Overall, conversion surgery should be positively considered as a treatment option in the era of targeted therapies, with increasing response rates.

In this cohort, among the factors obtained at diagnosis, normal BMI (*p* = 0.034) and either HER2+, MSI-H, or dMMR (*p* = 0.016) were significantly associated with a favorable prognosis in multivariable analysis. In contrast, a metastatic pattern was not significantly associated with prognosis, nor were comorbidities. This is in agreement with results from previous studies that demonstrate no survival difference between initial metastatic sites [15,21,22]. Favorable long-term survival in patients with multiple metastases in our results is unreasonable, and this may be because patients with multiple metastases who received conversion surgery were highly responsive to chemotherapy; only patients whose multiple metastatic tumors regressed very well after chemotherapy could be selected for surgery, and thus their great chemosensitivity might have led to favorable long-term survival. Accordingly, patients with initially unresectable metastases showed longer survival than patients with technically resectable metastasis when an R0 resection was achieved in the CONVO-GC-1 study [17]. Taken together, conversion surgery could be beneficial regardless of disease burden at diagnosis, including tumor size, biopsy histology, metastatic pattern, and CEA level, but BMI and molecular markers of targeted therapies are critical for selecting patients for conversion surgery.

Normal BMI at diagnosis as well as the receipt of targeted therapy were significant prognostic factors in the multivariable analysis, which included factors identified after chemotherapy. CEA level before surgery and objective response to chemotherapy were significantly associated with survival in the univariable analysis, but they lost significance in the multivariable analysis. However, the significance of CEA level (*p* = 0.055) was very close to the threshold, and it may have been attenuated by its correlation with targeted therapy—patients who received targeted therapies showed a trend of lower CEA levels (Figure S7). In line with this, the change in CEA level was significantly and independently associated with prognosis in a previous study of conversion surgery [20]. The chemotherapy response of metastatic sites was also an independent prognostic factor in the same study, but it was not separately evaluated in our study. Five or more cycles of preoperative chemotherapy did not extend survival compared to three or four cycles, supporting the idea that the four cycles of chemotherapy in AIO-FLOT trials would be enough for conversion to surgery [16,18].

In the multivariable analysis of significant prognostic factors identified before and after conversion surgery, normal BMI, targeted therapy, ypT stage, and peritoneal cytology retained their significance. Tumor size in the surgical specimen was a prognostic factor with a greater hazard ratio than initial tumor size (Table 2), implying that disease burden after chemotherapy is more significant than the initial disease burden. Although ypT and ypN stages were significantly associated with each other (Spearman rank correlation test, *R* = 0.59, *p* < 0.001), we included both factors in the multivariable analysis due to their clinical significance. The greater importance of the ypT stage is also noted in its greater hazard ratio, even though it is more divided into five stages than the ypN stage which is divided into four. A favorable prognostic association of partial (versus total) gastrectomy is consistent with a previous study [23], although it was excluded from our multivariable analysis due to a significant correlation with the ypT stage.

Our study has strengths due to its wealth of results from molecular biomarkers—such as HER2, MSI, MMR, and PD-L1—and the substantial number of patients who were treated with targeted agents. Yet, some limitations of our study have to be considered. It is a retrospective study conducted in a single institution, and a limited number of patients with specific features might hinder the identification of significant prognostic factors—for example, only seven patients were EBV-positive, nine patients were PD-L1-negative, 17 patients did not receive postoperative chemotherapy, and 19 patients had liver metastasis. In a previous study with a more balanced number of patients treated with (N = 80) or without (N = 42) postoperative chemotherapy, it was an independent prognostic factor [22].

## 5. Conclusions

In conclusion, BMI at diagnosis and use of targeted agents or ICIs for specific biomarkers are important prognostic factors for successful conversion surgery. With the introduction of biomarker-specific targeted agents such as MET inhibitor, HER2 inhibitor, Trastuzumab deruxtecan and ICIs for MSI-H, it is reasonable to foresee that the proportion of patients who will benefit from conversion surgery will increase in the near future. In addition, with powerful biologic agents for appropriate biomarkers, neoadjuvant-biomarker-driven trials should be actively pursued.

## Figures and Tables

**Table 1:** Clinicopathological characteristics of the enrolled patients.

Characteristics	Number of Patients
Total	118
Age, median, years (range)	56.5 (32–82)
Sex (%)	
Male	88 (74.6)
Female	30 (25.4)
Comorbidities (%)	
Hypertension	29 (24.6)
Diabetes mellitus	23 (19.5)
Ischemic heart disease	5 (4.2)
Other	15 (12.7)
Body mass index, median (range)	
Before chemotherapy	23.0 (16.0–34.6)
Before surgery	24.0 (15.5–36.0)
Tumor size, median, cm (range)	
Initial (CT)	7.0 (1.5–15.0)
Initial (EGD)	6.0 (1.5–15.0)
Surgical specimen	3.6 (0–18)
Metastasis (%)	
Peritoneum	63 (53.4)
Liver	19 (16.1)
Distant lymph node	36 (30.5)
Multiple	21 (17.8)
Chemotherapy regimen (%)	
Cytotoxic agents only	73 (61.9)
HER2 inhibitor ± Cytotoxic agents	21 (17.8)
ICI ± HER2 inhibitor ± Cytotoxic agents	18 (15.3)
MET/VEGFR2 inhibitor ± Cytotoxic agents	6 (5.1)
Pathologic stage (%)	
ypT0N0	21 17.8)
ypT0N1	2 (1.7)
ypT0N2	1 (0.8)
ypT1N0	13 (11)
ypT1N1	2 (1.7)
ypT1N2	2 (1.7)
ypT2N0	7 (5.9)
ypT2N1	5 (4.2)
ypT2N3	1 (0.8)
ypT3N0	12 (10.2)
ypT3N1	6 (5.1)
ypT3N2	9 (7.6)
ypT3N3	8 (6.8)
ypT4N0	5 (4.2)
ypT4N1	4 (3.4)
ypT4N2	5 (4.2)
ypT4N3	15 (12.7)

CT, computed tomography; EGD, esophagogastroduodenoscopy; ICI, immune checkpoint inhibitor; ypTN, pathologic tumor and nodal classifications after preoperative therapy.

**Table 2:** Univariable Cox proportional hazards regression analysis of overall survival.

Features	Number of Patients	HR	95% CI	*p*-Value
Tumor size (cm)				
Initial (CT)	117	1.11	1.00–1.22	0.0441
Initial (EGD)	90	1.12	0.99–1.26	0.0666
Surgical specimen	118	1.16	1.09–1.23	<0.0001
CEA (ng/mL)				
Before chemotherapy	105	1.00	0.99–1.00	0.3389
Before surgery	107	1.05	1.01–1.09	0.0191
ypT stage	118	1.92	1.43–2.58	<0.0001
ypN stage	118	1.88	1.45–2.43	<0.0001

HR, hazard ratio; CI, confidence interval; CT, computed tomography; EGD, esophagogastroduodenoscopy; CEA, carcinoembryonic antigen; ypTN, pathologic tumor and nodal classifications after preoperative therapy.

## Data Availability

The data are not publicly available due to privacy.

## Supplementary Materials

The following supporting information can be downloaded at: https://www.mdpi.com/article/10.3390/biomedicines11113097/s1, Table S1: Clinicopathological characteristics of the enrolled patients; Table S2: Chemotherapy regimen administered just before the conversion surgery; Table S3: Recurred locations after conversion surgery (multiple sites counted for each patient); Table S4: *p*-Value from Pearson’s Chi-squared tests between post-operative pathological findings; Figure S1: Association of baseline features with overall survival (OS); Figure S2: Association of initial BMI with overall survival (OS) in patients with (a) HER2- and (b) HER2+ tumors; Figure S3: Association of metastatic sites with overall survival (OS); Figure S4: Association of post chemotherapy features with overall survival (OS); Figure S5: Association of post operative features with overall survival (OS). (a) Operational (OP) method comparing partial vs total gastrectomy. (b) Pathological diffrentiation status comparing moderately diffrentiated adenocarcinoma (MD) vs poorly differentiated adenocarcinoma or signet ring cell carcinoma (PD/SRC); Figure S6: Association between objective response to chemotherapy and CEA level before operation; Figure S7: Association between receipt of targeted therapy and CEA level before operation.
