# Supplementary material for: The Clinical Implication of Conversion Surgery in Patients with Stage IV Gastric Cancer Who Received Systemic Chemotherapy
Source: Biomedicines. 2023 Nov 20;11(11):3097. doi: 10.3390/biomedicines11113097 (PMC10669208; doi:10.3390/biomedicines11113097)
Supplement: Supplementary file 1 [file biomedicines-11-03097-s001.zip › biomedicines-2667317-supplementary.pdf]

Table S1. Clinicopathological characteristics of the enrolled patients

Characteristics	Number of patients
**Biopsy pathology (%)**	
Well/Moderately differentiated adenocarcinoma	38 (32.2)
Poorly differentiated adenocarcinoma	59 (50)
Signet ring cell/Poorly cohesive carcinoma	20 (16.9)
Adenocarcinoma with neuroendocrine differentiation	1 (0.8)
**HER2 (%)**	
negative	90 (76.3)
positive	26 (22.0)
**EBV (%)**	
negative	95 (80.5)
positive	7 (5.9)
**PD-L1, median, CPS (range)**	2 (0-95)
**MSI (%)**	
MSI-H	4 (3.4)
MSS	42 (35.6)
**MMR (%)**	
Deficient	4 (3.4)
Intact	56 (47.5)
**Cycles of the preoperative chemotherapy regimen, median (range)**	7 (3-35)
**Best response to chemotherapy (%)**	
Complete response	5 (4.2)
Partial response	84 (71.2)
Stable disease	29 (24.6)
**CEA, median, ng/mL (range)**	
Before chemotherapy	1.6 (0.2-1000)
Before surgery	2.0 (0.4-64.9)
**Follow-up period, median, day (range)**	
From diagnosis	1,046 (305-2,907)
From surgery	720 (53-2,335)

EBV, Epstein-Barr virus; MSI, microsatellite instability; MSS, microsatellite stable; MMR, mismatch repair; CEA, carcinoembryonic antigen.

Table S2. Chemotherapy regimen administered just before the conversion surgery

Regimens	Number of patients
**Cytotoxic**	**73**
XELOX(capecitabine+oxaliplatin)	68
FOLFOX(5-FU+leucovorin+oxaliplatin)	1
FOLFIRI(5-FU+leucovorin+irinotecan)	4
**HER2i**	**21**
XP(capecitabine+cisplatin)+Trastuzumab	17
XP+Trastuzumab+Pertuzumab	1
XELOX+Trastuzumab	1
Capecitabine+Her2 inhibitor	2
**Immune checkpoint inhibitor**	**18**
Pembrolizumab	4
XELOX+Pembrolizumab	9
XELOX+Trastuzumab+Pembrolizumab	2
XELOX+Nivolumab	3
**METi**	**4**
**VEGFR2i**	**2**

Table S3. Recurred locations after conversion surgery (multiple sites counted for each patient)

Locations	Number of patients
Peritoneum	20
Distant lymph nodes	13
Ovary, ureter or bladder	10
Gastrectomy site, duodenal or jejunal stump	8
Liver	7
Pancreas or adrenal gland	5
Abdominal wall	4
Colon or rectum	3
Unspecified retroperitoneum	3
Subhepatic space, gall bladder, or common bile duct lymph node	3
Bone	2
Cerebrospinal fluid	1

Table S4. P-value from Pearson's Chi-squared tests between post-operative pathological findings

	Lymphatic invasion	Venous invasion	Perineural invasion
**ypT stage**	<0.0001	0.2199	<0.0001
**ypN stage**	<0.0001	0.01343	0.0003

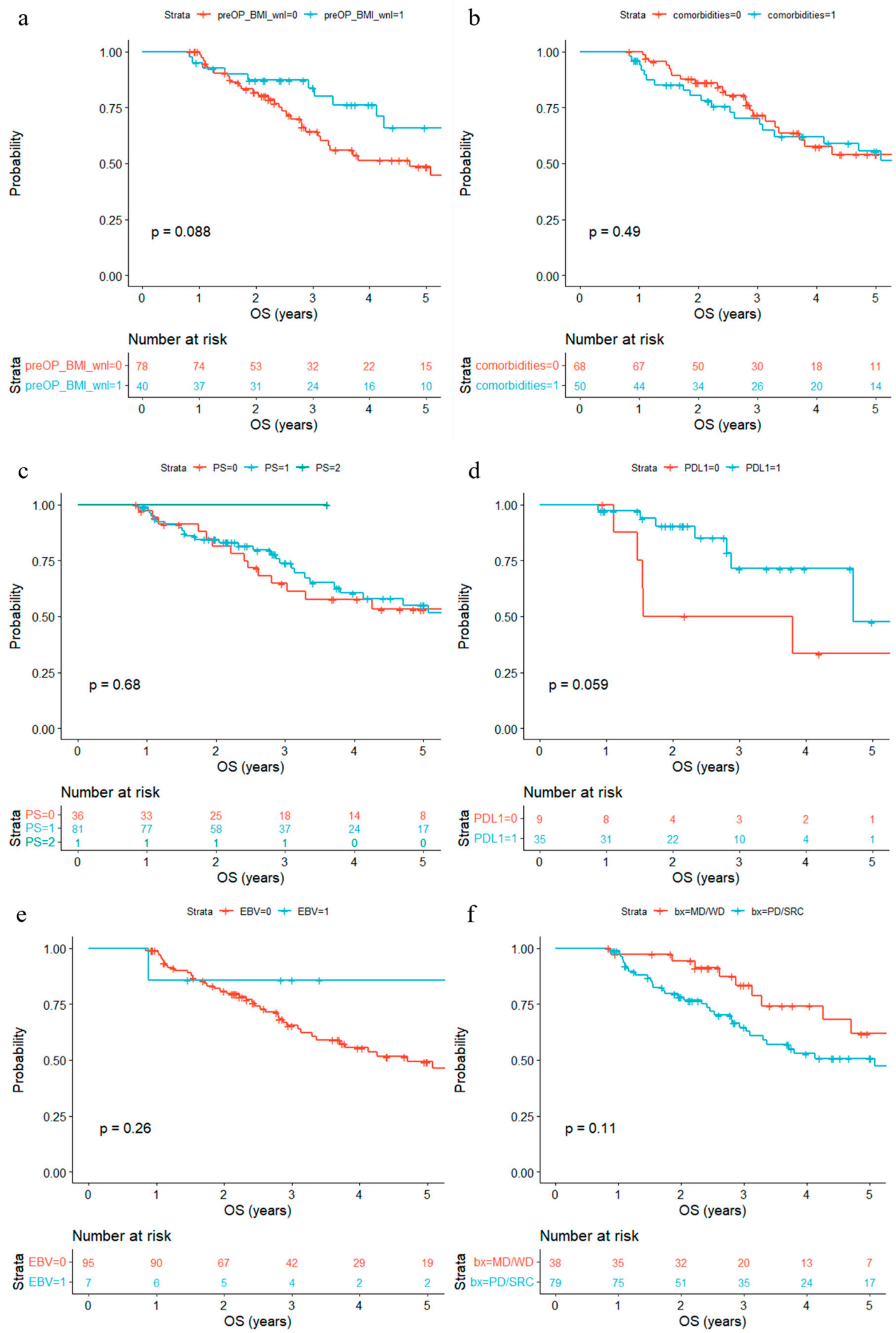

Figure S1. Association of baseline features with overall survival (OS)

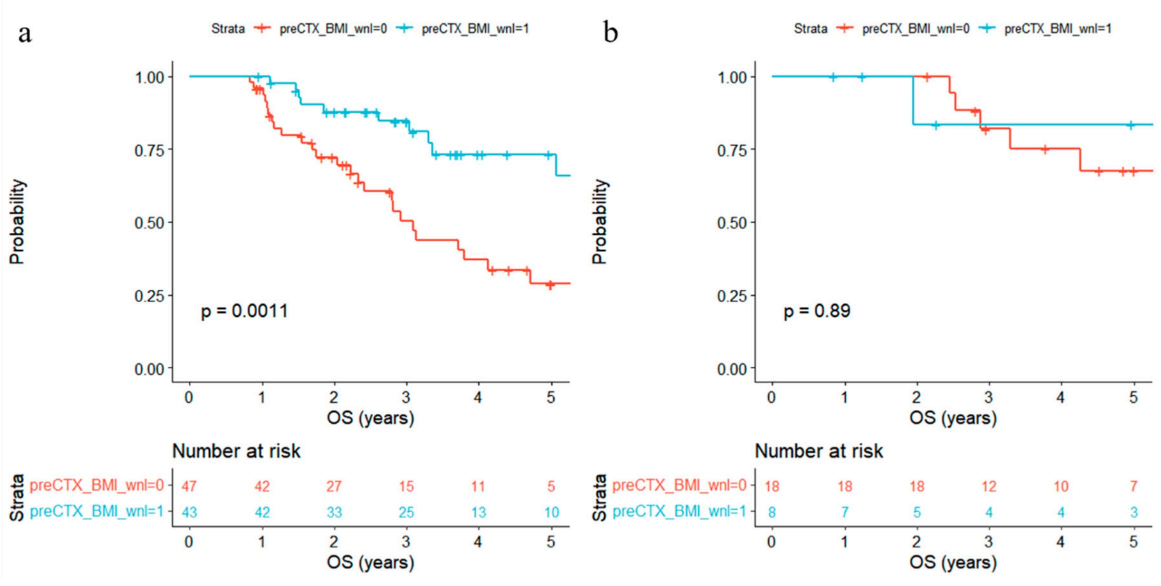

Figure S2. Association of initial BMI with overall survival (OS) in patients with (a) HER2- and (b) HER2+ tumors.

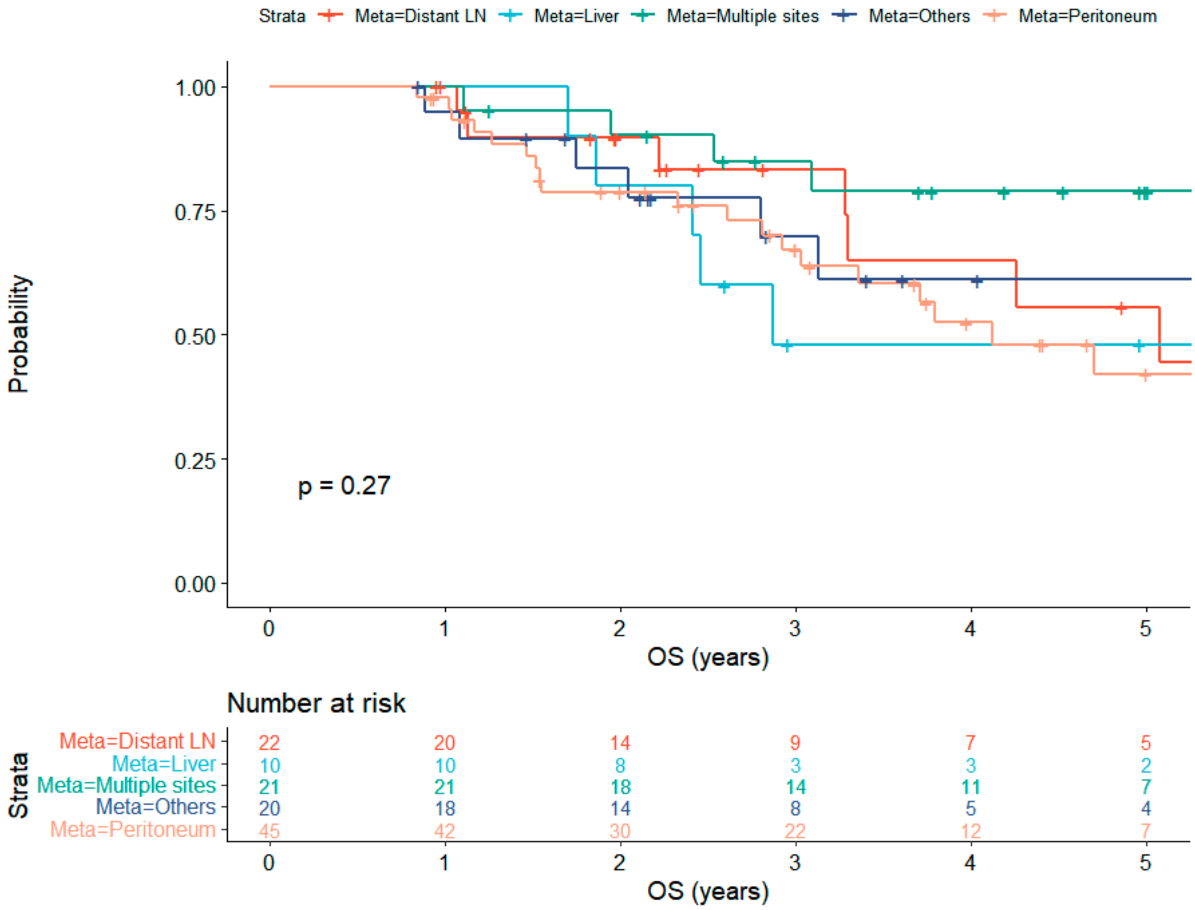

Figure S3. Association of metastatic sites with overall survival (OS)

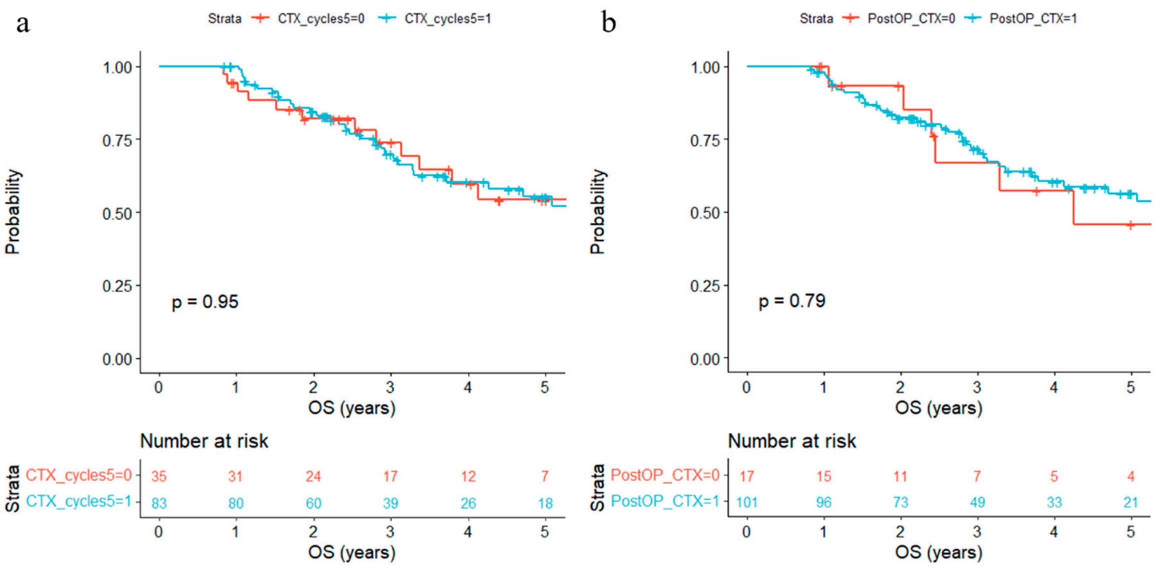

Figure S4. Association of post chemotherapy features with overall survival (OS)

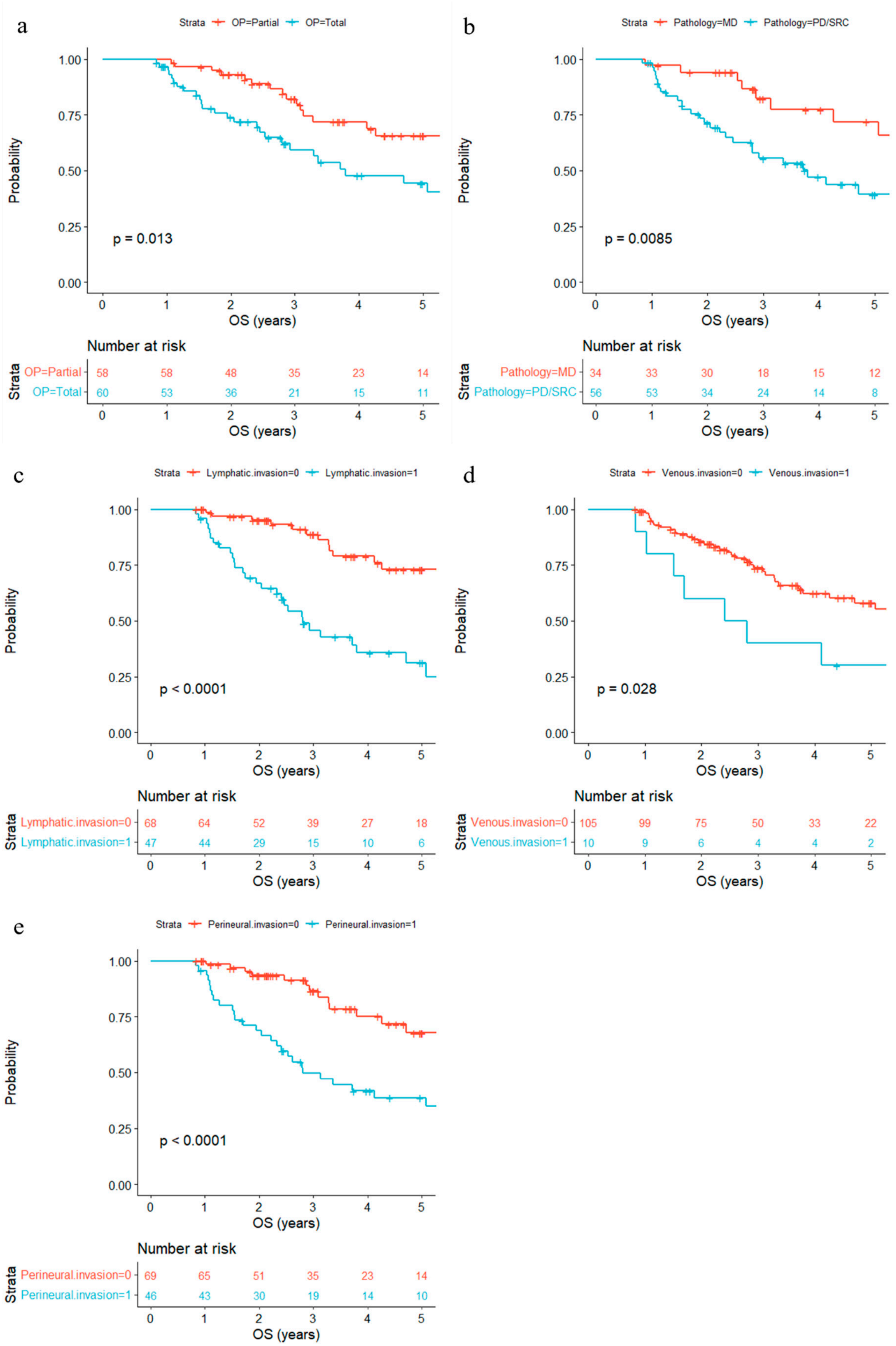

Figure S5. Association of post operative features with overall survival (OS). (a) Operational (OP) method

comparing partial vs total gastrectomy. (b) Pathological diffrentiation status comparing moderately diffrentiated adenocarcinoma (MD) vs poorly differentiated adenocarcinoma or signet ring cell carcinoma (PD/SRC).

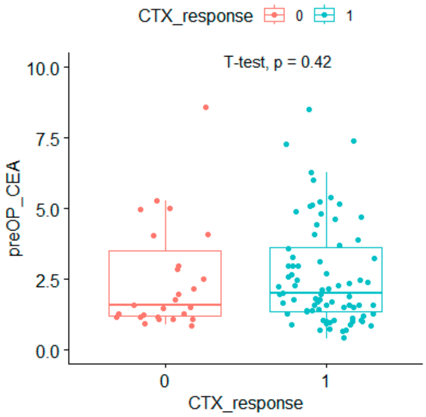

Figure S6. Association between objective response to chemotherapy and CEA level before operation

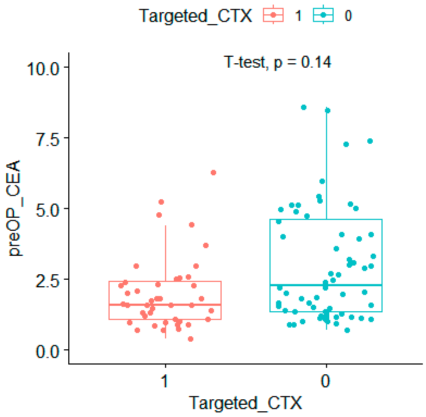

Figure S7. Association between receipt of targeted therapy and CEA level before operation
